# The lateral reticular nucleus; integration of descending and ascending systems regulating voluntary forelimb movements

**DOI:** 10.3389/fncom.2015.00102

**Published:** 2015-08-05

**Authors:** Bror Alstermark, Carl-Fredrik Ekerot

**Affiliations:** ^1^Department of Integrative Medical Biology, Section of Physiology, Umeå UniversityUmeå, Sweden; ^2^Department of Experimental Medical Science, University of LundLund, Sweden

**Keywords:** interneurons, propriospinal neurons, motoneurons, lateral reticular nucleus, cerebellum, motor control, efferent copy, internal feedback

## Abstract

Cerebellar control of movements is dependent on mossy fiber input conveying information about sensory and premotor activity in the spinal cord. While much is known about spino-cerebellar systems, which provide the cerebellum with detailed sensory information, much less is known about systems conveying motor information. Individual motoneurones do not have projections to spino-cerebellar neurons. Instead, the fastest route is from last order spinal interneurons. In order to identify the networks that convey ascending premotor information from last order interneurons, we have focused on the lateral reticular nucleus (LRN), which provides the major mossy fiber input to cerebellum from spinal interneuronal systems. Three spinal ascending systems to the LRN have been investigated: the C3-C4 propriospinal neurones (PNs), the ipsilateral forelimb tract (iFT) and the bilateral ventral flexor reflex tract (bVFRT). Voluntary forelimb movements involve reaching and grasping together with necessary postural adjustments and each of these three interneuronal systems likely contribute to specific aspects of forelimb motor control. It has been demonstrated that the command for reaching can be mediated via C3-C4 PNs, while the command for grasping is conveyed via segmental interneurons in the forelimb segments. Our results reveal convergence of ascending projections from all three interneuronal systems in the LRN, producing distinct combinations of excitation and inhibition. We have also identified a separate descending control of LRN neurons exerted via a subgroup of cortico-reticular neurones. The LRN projections to the deep cerebellar nuclei exert a direct excitatory effect on descending motor pathways via the reticulospinal, vestibulospinal, and other supraspinal tracts, and might play a key role in cerebellar motor control. Our results support the hypothesis that the LRN provides the cerebellum with highly integrated information, enabling cerebellar control of complex forelimb movements.

## Introduction

Cerebellar control of movements requires continuous information about descending motor commands and sensory information evoked by external events. Such information is provided by mossy fiber and climbing pathways (Ito, 1984). As to mossy fiber systems, much attention has been given to direct spino-cerebellar pathways that may provide information about information about internal copies of motor commands (Lundberg, 1971; Arshavsky et al., 1972, 1978; Alstermark and Isa, 2012; Fedirchuk et al., 2013; Azim and Alstermark, 2015), from receptors about external events (Stecina et al., 2013) and inhibition of self-evoked reafferent signals by movements (Hantman and Jessell, 2010). However, less focus has been given to indirect spino-cerebellar systems. In a companion *Perspective article* of this Frontier Research Topic, we compare direct and indirect spino-cerebellar systems (Jiang et al., 2015). Of particular interest are systems conveying spinal information via the lateral reticular nucleus (LRN). The LRN in the caudal brain stem is a major mossy fiber input from the spinal cord, projecting to cerebellar cortex and sending collaterals to the deep cerebellar nuclei (Matsushita and Ikeda, 1976; Dietrichs and Walberg, 1979; Ito et al., 1982). Importantly, the extensive collateral projections to the deep cerebellar nuclei from the LRN exert a direct excitatory effect on post-cerebellar descending motor pathways, and thus can quickly modulate motor control.

Three major ascending systems from the spinal cord to the LRN have been described in the cat: the bilateral ventral flexor reflex tract (bVFRT; Clendenin et al., 1974b; Ekerot, 1990b), the ipsilateral forelimb tract (iFT; Clendenin et al., 1974c; Ekerot, 1990a) and the C3-C4 propriospinal neurones (C3-C4 PNs; Illert and Lundberg, 1978; Alstermark et al., 1981a). However, while LRN input from the iFT and bVFRT have been previously investigated (Clendenin et al., 1974a,b,c; Ekerot, 1990a,b), the role of C3-C4 PN input, and how signals from all three ascending systems are integrated in the LRN, remains unknown.

The C3-C4 propriospinal system is of special interest because these neurones project not only to the LRN, but also to forelimb motoneurones (MNs), conveying both ascending and last-order premotor information (Illert and Lundberg, 1978; Alstermark et al., 1981a; Isa et al., 2006). The function of the C3-C4 PNs has been investigated in behavioral experiments, revealing a role in mediating the voluntary command for visually guided forelimb reaching in the cat (Alstermark et al., 1981b; Alstermark and Kümmel, 1990) and additionally for precision grip in the macaque monkey (Sasaki et al., 2004; Alstermark et al., 2011; Kinoshita et al., 2012). The C3-C4 PNs are characterized by monosynaptic excitation from cortico-, rubro-, tecto- and reticulospinal fibers as well as from cutaneous and muscle afferents in the forelimb nerves (Illert et al., 1978). In addition, all of these converging descending and sensory inputs can mediate disynaptic inhibition of C3-C4 PNs via feed-forward and feed-back inhibitory interneurones (Alstermark et al., 1984c). These excitatory and inhibitory inputs are integrated by C3-C4 PNs, which then excite or inhibit forelimb MNs (Illert et al., 1977; Alstermark et al., 1984a,b; Alstermark and Sasaki, 1985).

In addition to targeting MNs, C3-C4 PN output bifurcates and ascends to the LRN, potentially providing the cerebellum with efference copy, now often referred to as internal feedback. Such information about the ongoing reaching movement may allow the cerebellum to quickly modify the descending motor command via the rubro- and reticulospinal systems (Alstermark et al., 1981a). This idea was recently supported by a combined electrophysiological, optogenetic and behavioral study in the mouse, in which the ascending branch from V2a PNs to the LRN could be selectively activated (Azim et al., 2014). These authors, found that photoactivation of V2a PN terminals evoked strong activation of LRN neurons that caused errors in reaching, but not grasping.

This study aims to investigate convergence of the iFT, bVFRT and C3-C4 propriospinal systems onto individual LRN neurones in the cat in order to clarify how ascending information from functionally different circuits is processed in the LRN. We find a minority of LRN neurones with input from only individual ascending systems, and a majority of neurones with convergent input from two or all three systems, suggesting that subpopulations of LRN neurones integrate ascending premotor information from the forelimb to enable cerebellar modulation of ongoing movement. A preliminary report has been presented (Alstermark and Ekerot, 2013).

## Materials and Methods

All procedures were approved by the local ethical committees (at the University of Göteborg and University of Lund) and were in accordance with Swedish regulations on animal experimentation.

### Preparation

The results were obtained from nine adult cats (body weight 2.5–3.1 kg). Intial anesthesia was ketamine/ether followed by α-chloralose (80 mg/kg). Additional doses of α-chloralose were administered during the course of the experiment. The criteria for adequate depth of anesthesia were the persistence of miotic pupils, stable blood pressure and respiratory rate and absence of the withdrawal reflex to noxious stimulation. The paralytic gallamine triethiodide (Flaxedil) was administered, and pneumothorax and artificial ventilation were performed to minimize movement artifacts. After paralysis, the criteria for adequate anaesthesia were miotic pupils and stable blood pressure, even with painful stimuli. Rectal temperature was maintained at 36–38°C, and arterial blood pressure and expiratory CO_2_ (4.0%) were monitored continuously. Blood pressure was maintained above 80 mmHg in all experiments. Laminectomy was performed to expose the spinal segments C1-C8 and Th11–13. The superficial (SR) and deep radial (DR) nerves on both sides were dissected and mounted on cuff electrodes. Craniotomy was performed to expose the caudal brain stem, the cerebellum and the cortex overlying nucleus ruber (NR). The experiments were terminated by a lethal of pentobarbital sodium.

### Stimulation and Recording

**Figure 1:**
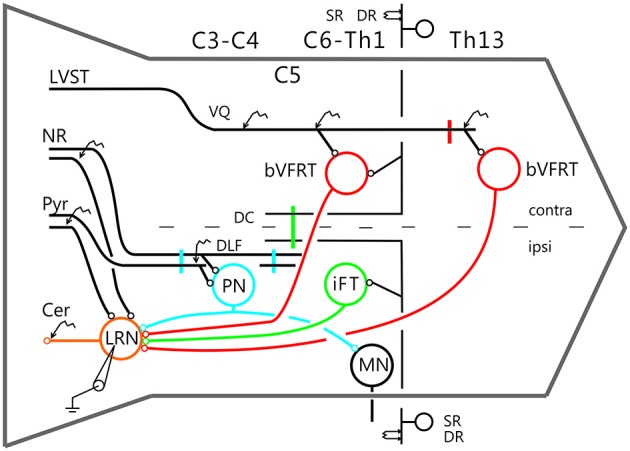
**Schematic outline of the experimental set-up**. Intracellular recordings were made from antidromically identified neurones in the lateral reticular nucleus (LRN) by electrical stimulation in the cerebellar (Cer) white matter. Possible convergence from bifurcating C3-C4 propriospinal neurones (C3-C4 PNs), which project to LRN neurones and motoneurones (MNs) in the forelimb segments C6-Th1; ipsilateral forelimb tract (iFT); and the bilateral flexor reflex tract (bVFRT) was tested in LRN neurones using electrical stimulation. C3-C4 PNs were activated from fibers in the contralateral pyramid (Pyr) and nucleus ruber (NR) after transection of these fibers in the dorsolateral funiculus (DLF) in C5. A control lesion was made in C2 to eliminate cortico-and rubrospinal input to C3-C4 PNs. C3-C4 PNs could also be activated by stimulation of cortico- and rubrospinal fibers in the DLF in the C3-C4 segments following DLF transections in both C5 and C2. iFT neurones were activated by stimulation of primary afferents in the ipsilateral superficial (SR) and deep radial (DR) nerves (SR and DR) after transection of the dorsal column (DC) in C5 to eliminate afferent input to the more rostrally located C3-C4 PNs. bVFRT neurones were activated from the contralateral SR and DR nerves after a DC transection in C5 and from the lateral vestibulospinal tract (LVST) via stimulation in the contralateral ventral quadrant (cVQ) in C4 or C6. In order to prevent activation of bVFRT neurones in the lumbar segments by LVST stimulation, the contralateral spinal cord was transected at Th13. The lumbar bVFRT neurons were activated by stimulation of the LVST caudal to lesion in in Th13.

The experimental setup is shown in Figure 1. Corticofugal fibers were stimulated in the contralateral pyramid (Pyr) 3–4 mm rostral to obex at the caudal end of the 4th ventricle, rubrospinal fibers in the contralateral NR (NR; Horsley-Clarke coordinates A3, L1.5, H-2.5), and fibers in the lateral vestibulospinal tract (LVST) were stimulated in the contralateral ventral quadrant (coVQ) either in C4 or C6 using monopolar tungsten electrodes, and in Th13 using bipolar silver electrodes. To restrict the activation of the bVFRT to the cervical cord, the dorsal columns (DC) were removed and a hemisection contralateral to the recording side was performed in Th11. Intracellular recording was made with glass microelectrodes filled with 2 M potassium acetate (tip diameter 1.0–2.0 μm, impedance 2–5 MΩ). LRN neurones were identified via antidromic activation from the ipsilateral cerebellar white matter dorsal to the interpositus nucleus at a depth of 6 mm below the cerebellar surface (insertion point of the electrode: 1–2 mm caudal to the primary fissure at a laterality of 4 mm). The arrival of the incoming volley to the LRN neurons was recorded by silver ball electrode at the surface near the patch used for the intracellular recordings. The stimulating and recording sites were verified histologically. The position of NR was also verified by recording the antidromic response followed by stimulation of the rubrospinal axons in Th13.

### Delineation of Spinal Systems Ascending to the LRN

In order to independently characterize the influence of each ascending system on the LRN, we took advantage of their differences in neuronal input and spinal cord location. The C3-C4 PN system is activated by stimulation of cortico- and rubrospinal fibers. It has been demonstrated that the vast majority (84%) of the C3-C4 PNs with projection to motoneurons have ascending projection to the LRN (Alstermark et al., 1981a). We identified C3-C4 PN effects in the LRN via transection of cortico- and rubrospinal fibers in the dorsolateral funiculus (DLF) in C5 (sparing the input to the C3-C4 PNs, while interrupting input to the more caudal (iFT system). In three experiments the DLF was transected in C2 to interrupt the input both to C3-C4 PNs and more caudal systems. In addition, when both C5 and C2 DLF lesions were performed, the C3-C4 PNs could be synaptically activated in isolation via stimulation of cortico- and rubrospinal fibers in the DLF of the C3 segment. The isolated strip of DLF was stimulated using a monopolar tungsten electrode. Threshold for evoking the DLF volley was 10 μA. The threshold for current spread to the nearby dorsal column was checked by recording from the SR and DR nerves and was usually approximately 500 μA.

The iFT-system is characterized by strong activation from forelimb nerves (Clendenin et al., 1974b; Ekerot, 1990b). In order to restrict activation to iFT neurones in the forelimb segments (C6-Th1), the dorsal column (DC) was transected in C5.

The bVFRT neurones are monosynaptically activated from the LVST and have large, often bilateral, receptive fields (Clendenin et al., 1974c; Ekerot, 1990a). Effects in the LRN mediated via the subcomponents of the bVFRT-system were restricted by transection of the contralateral LVST in Th13 and the primary afferents in the dorsal column in C5. Cervical bVFRT neurons were activated by stimulation of the LVST in the cVQ in C4 or C6. Lumbar bVFRT neurons were activated by stimulation of the contralateral LVST in L2 caudal to transection in Th13.

## Results

Intracellular recordings were made from 113 LRN neurons identified via antidromic activation from the ipsilateral cerebellar white matter as described in the methods.

### Effects in LRN Neurons Evoked from Pyramidal and Rubrospinal Tracts

In order to examine monosynaptic excitatory effects of descending cortico- and rubro-spinal tracts on neurons in the LRN, and disynaptic excitation and inhibition mediated via C3-C4 PNs, recording was assessed following DLF transections of descending tracts in C2 or C5. The rostral C2 DLF transection eliminates the inputs from cortico- and rubrospinal fibers to the C3-C4 PN, whereas the C5 DLF transection spares this input, but eliminates it to more caudal spinal levels. Note, that after the C2 DLF transection both the input from pyramid and NR to the LRN is intact.

**Figure 2:**
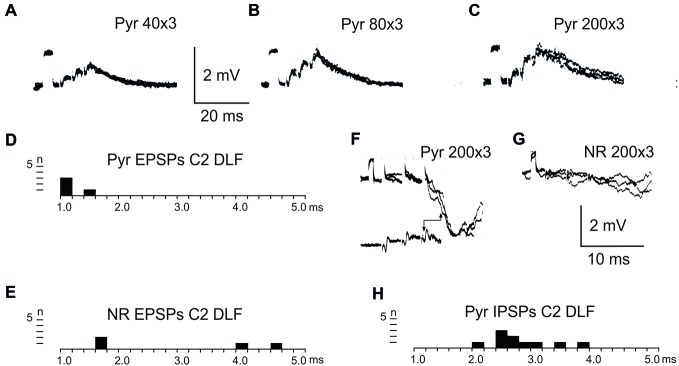
**Pyramidal and rubral effects after C2 DLF transection. (A–C)**, intracellular recordings from a LRN neuron when applying a train of three stimuli to the contralateral pyramid. **(D,E)**, distribution of excitatory postsynaptic potential (EPSP) latencies measured from the incoming volley evoked by stimulation in the contralateral pyramid and NR. **(F,G)**, intracellular from another LRN neuron showing disynaptic pyramidal IPSPs **(F)** but no IPSPs evoked by NR stimulation **(G)**. In inhibitory postsynaptic potentials (IPSPs; **F**) is shown the cord dorsum recording of the pyramidal volleys below the intracellular traces. Recording of the volleys was made rostral to the C2 DLF transection. The two arrow heads indicate the latency measurement from the positive/negative phase of the triphasic pyramidal volley to the IPSP onset. **(H)**, distribution of IPSP latencies measured from the incoming volley evoked by stimulation in the contralateral pyramid.

Figure 2 shows recordings from two LRN neurones after a C2 DLF transection. The contralateral pyramid was stimulated by a train of three volleys at different strengths (Figures 2A–C). Small monosynaptic excitatory postsynaptic potentials (EPSPs) could be evoked with a threshold below 40 μA (Figure 2A). The amplitude increased when the current stimulus intensity was raised to 80 and 200 μA (Figures 2B,C). The longer EPSP duration with 200 μA is likely due to activation slower conducting corticoreticular fibers. Monosynaptic pyramidal EPSPs were observed in 25% of the neurones. Monosynaptic rubral EPSPs after C2 DLF transection was observed in 10% of the neurones (not illustrated). Lack of disynaptic cortico- or rubral EPSPs were observed in all of the 40 cells tested. The segmental latencies are shown in Figures 2D,E. In contrast, disynaptic pyramidal inhibitory postsynaptic potentials (IPSPs) could be elicited after C2 DLF transection, but at lower frequency (7/19 neurones) compared to before the lesion (33/66 neurones). One example of pyramidal disynaptic IPSPs is shown in Figure 2F. Rubral IPSPs were lacking following C2 DLF transection (0/21 neurones) and one example is shown in Figure 2G, which is taken from the same LRN cell as in (Figure 2F). These data reveal monosynaptic excitation of LRN neurons by cortico- and rubro-reticular fibers, and disynaptic pyramidal inhibition, but not from NR.

**Figure 3:**
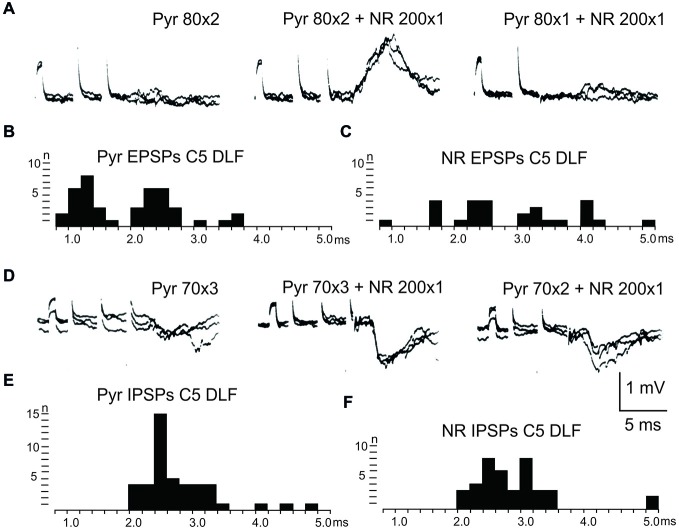
**Convergence of pyramidal and rubral effects after C5 DLF transection. (A)**, intracellular recordings from a LRN neuron showing spatial facilitation disynaptic EPSPs when applying a train of two stimuli to the contralateral pyramid alone (left panel), combined with conditioning single stimulus to the contralateral NR (middle panel) and when the second pyramidal stimulus was removed but the conditioning rubral stimulation remained (right panel). Note that the rubral conditioning stimulation was given synchronously with the second pyramidal stimulation. **(B,C)**, distribution of EPSP latencies measured from the incoming volley evoked by stimulation in the contralateral pyramid and NR, respectively. Measurements were made from the last effective volley of the train in this and the following latency histograms. **(D)**, intracellular recording from another LRN neuron showing spatial facilitation of IPSPs when applying a train of three stimuli to the contralateral pyramid alone (left panel), combined with conditioning single stimulus to the contralateral NR (middle panel) and when the third pyramidal stimulus was removed but the conditioning rubral stimulation remained (right panel). **(E,F)**, distribution of IPSP latencies measured from the incoming volley evoked by stimulation in the contralateral pyramid and NR, respectively.

After C5 DLF transection of cortico-and rubrospinal fibers, leaving descending connections with the LRN neurons and C3-C4 PNs, but not with interneurons in the forelimb segments, monosynaptic EPSPs and disynaptic and late EPSPs and IPSPs could be evoked in the LRN from the contralateral pyramid and NR. The expected minimal disynaptic linkage of pyramidal and rubral EPSPs and IPSPs to LRN via C3-C4 PNs is 2.1 ms, and the maximal linkage is 2.9 ms (based on a conduction velocity of 60 m/s for the corticospinal fibers and 26 m/s for the ascending branch of the C3-C4 PNs; Alstermark et al., 1981a). Disynaptic pyramidal EPSPs were found in 28% (19/67 neurones) and IPSPs in 55% (36/66 neurones). Disynaptic rubral EPSPs were observed in 16% (11/69 neurones) and IPSPs in 46% (32/69 neurones). The higher frequencies of the IPSPs most likely reflect the fact that the membrane potential decreased after electrode impalement of the LRN cells, making it easier to record IPSPs than EPSPs. Figure 3 shows spatial facilitation of disynaptic pyramidal and rubral EPSPs and IPSPs in LRN neurones following stimulation of cortico- and rubrospinal fibers with a short train of 2–3 volleys after C5 DLF transection (Figures 3A,B), as well as the distribution of latencies (measured from the effective stimulation pulse) in Figures 3C,D.

These findings strongly suggest that disynaptic pyramidal and rubral excitation and inhibition in LRN neurones can be mediated by excitatory, respectively inhibitory C3-C4 PNs. The disynaptic pyramidal IPSPs that remain after the C2 DLF transection are presumably mediated via reticulospinal neurones. It is worth noting that all rubral IPSPs were mediated via spinal neurones located caudal to C2 (cf. Figures 2 and 3).

**Figure 4:**
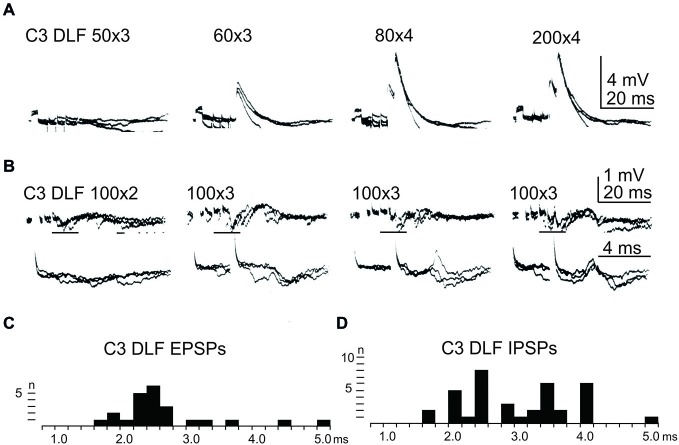
**Effects evoked from the isolated C3 DLF segment. (A)**, intracellular recordings from a LRN showing disynaptic EPSPs when applying a train of three stimuli to the isolated C3 DLF. **(B)**, intracellular recordings from a LRN showing disynaptic EPSPs and IPSPs when applying a train of three stimuli to the isolated C3 DLF. The same traces are shown at a slow (upper panels) and fast (lower panels) sweep speed. Note, the lower panels only show part of the upper records, indicated by the horizontal black line. **(C,D)**, distribution of EPSP respectively IPSP latencies measured from the incoming volley evoked by stimulation in the isolated C3 DLF segment.

In order to further delineate the LRN effects of C3-C4 PN activation, the DLF was transected in C2 and C5. The isolated C2 to C5 strip of DLF was then stimulated electrically using a monopolar tungsten electrode. Figure 4 shows that stimulation of cortico- and rubro-spinal fibers in the C3 DLF evoked disynaptic EPSPs and IPSPs in LRN neurones with latencies within the expected disynaptic range of 1.6–2.4 ms. These data further indicate that the disynaptic LRN effects of cortico- and rubro-spinal excitation are mediated by PNs located in the C3 to C4 segments.

After combined C2 and C5 DLF lesions, it was common to find a mixture of excitation and inhibition in many of the LRN neurones following cortico- and rubro-spinal fiber stimulation. Disynaptic EPSPs evoked by a train of stimuli in the C3 DLF are shown in Figure 4A. In another cell illustrated in Figure 4B, a mixture of EPSPs and IPSPs were evoked at the same latency when the membrane potential remained virtually unchanged, shown in Figures 4C,D. It can be more easily observed in the expanded sweeps (lower records). These results strongly suggest that a subpopulation of LRN neurones receives mixed input from excitatory and inhibitory C3-C4 PNs.

### Effects in LRN Neurons Evoked from the iFT

**Figure 5:**
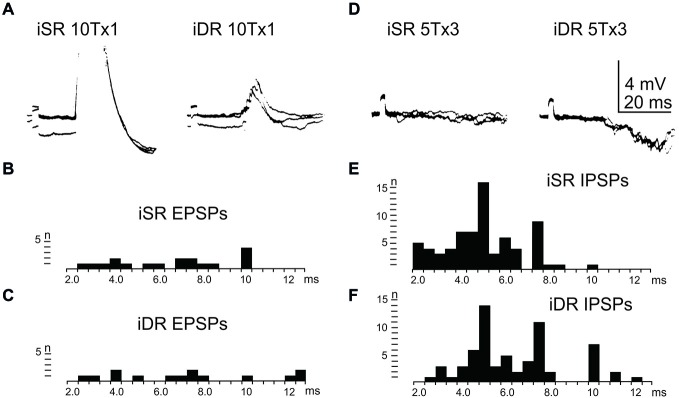
**Effects evoked via the iFT. (A)**, intracellular recordings from a LRN neuron when stimulating ipsilateral forelimb nerves, SR radial (iSR) and deep radial (iDR), at 10 times threshold. **(B,C)**, distribution of EPSP latencies by electrical stimulation of the iSR respectively iDR nerves. **(D)**, intracellular recordings from another LRN neuron when stimulating the iSR and iDR nerves. **(E,F)**, distribution of IPSP latencies by electrical stimulation of the iSR respectively iDR nerves.

In order to assess the influence of iFT neurones onto the LRN, we first confirmed earlier findings by Clendenin et al. (1974a,c) and Ekerot (1990a) that short latency excitation and inhibition in LRN neurones could be evoked by stimulation of ipsilateral forelimb afferents (see Figure 1). The effects of iFT activation are shown from two different LRN neurones in Figures 5A,D respectively for the cutaneous (iSR) and muscle forelimb (iDR) nerves. Latencies of EPSPs (Figures 5B,C) and IPSPs (Figures 5E,F) below 4 ms are compatible with a disynaptic transmission.

### Effect in LRN Neurons Evoked from the Cervical bVFRT

**Figure 6:**
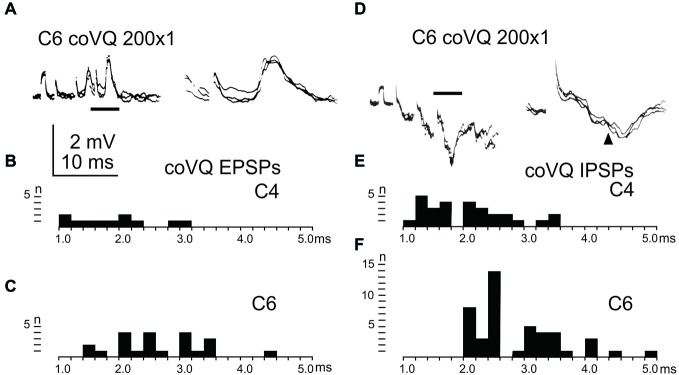
**Effects evoked via the cervical bVFRT. (A)**, intracellular recordings from a LRN neuron showing disynaptic EPSPs when stimulating the LVST with a train of three stimuli in the (coVQ) in the C6 segment. The right panel was taken at a higher sweep speed and is expanded from the section indicated by the horizontal line in the left panel. **(B,C)**, distribution of EPSP latencies by electrical stimulation of the LVST in the coVQ in C4 respectively C6. **(D)**, intracellular recordings from a LRN neuron showing disynaptic IPSPs when stimulating the LVST with a train of three stimuli in the (coVQ) in the C6 segment. The right panel was taken at a higher sweep speed and is expanded from the section indicated by the horizontal line in the left panel. **(E,F)**, distribution of IPSP latencies by electrical stimulation of the coVQ in the C4 and C6 segments, respectively.

We also tested the effects of cervical bVFRT neurones on the LRN by stimulation of the lateral vestibulospinal tract in the contralateral (coVQ) in C4 or C6. We confirmed earlier findings by Clendenin et al. (1974b) and by Ekerot (1990b) that disynaptic excitation and inhibition in the LRN could be evoked by cervical bVFRT stimulation, as shown in two different LRN neurones in Figures 6A,D. Figure 6A illustrates disynaptic EPSPs evoked by the second and third volley (shown at higher sweep speed in the right panel). Figure 6D illustrates disynaptic IPSPs evoked by the first volley alone. In the right panel, taken at higher sweep speed, a second IPSP can be observed at trisynaptic latency (arrowhead). Note the prolongation of the minimal latencies when stimulating in C6 compared to C4, as shown in Figures 6B,C,E,F, respectively. This is expected because the bVFRT neurons are located downstream of the stimulation site.

Recording was also made from LRN neurons when stimulating the LVST below the transection in Th13 in order to activate bVFRT neuron located in the lumbar segments (not illustrated). We confirmed the previous findings by Clendenin et al. (1974b) and Ekerot (1990b) that LRN neurons receive input from both excitatory and inhibitory lumbar bVFRT neurons as has also recently been demonstrated anatomically in the rat by Huma and Maxwell (2015). We provide data from LRN neurons with input from lumbar bVFRT below in Tables 1 and 2.

**Table 1:** **Distribution of monosynaptic excitation from pyramid among subpopulations of LRN neurons**.

System	Pyramid
Excit iFT	30% (6/20)
Inhib iFT	60% (12/20)
Excit cerv bVFRT	35% (6/17)
Inhib cerv bVFRT	53% (9/17)
Mono NR excitation	5% (1/20)
Excit lumb bVFRT	6% (1/17)
Inhib lumb bVFRT	12% (2/12)

*The frequency is given both in percentage and the number of tested cells within parenthesis*.

**Table 2:** **Distribution of disynaptic EPSPs and IPSPs from pyramid and nucleus ruber mediated via C3-C4 PNs among subpopulations of LRN neurons**.

LRN System	Pyramid		Nucleus Ruber	
	di Pyr EPSP	di Pyr IPSP	di NR EPSP	di NR IPSP
Exc iFT	35% (6/17)	16% (3/19)	72% (5/7)	21% (3/14)
Inh iFT	59% (10/17)	90% (17/19)	86% (6/7)	86% (12/14)
Exc cerv bVFRT	36% (5/14)	28% (5/18)	83% (5/6)	40% (4/10)
Inh cerv bVFRT	50% (7/14)	67% (12/18)	50% (3/6)	91% (10/11)
Monosyn Pyr	28% (5/18)	30% (6/20)	57% (4/7)	21% (3/14)
Monosyn NR	0% (0/18)	5% (1/20)	0% (0/8)	7% (1/15)
Exc lumb bVFRT	23% (3/13)	29% (4/14)	17% (1/6)	33% (3/9)
Inh lumb bVFRT	31% (4/13)	69% (11/16)	33% (2/6)	83% (10/12)

*The frequency is given both in percentage and the number of tested cells within parenthesis*.

### Location of LRN Neurones with Input from the Pyramid and NR

**Figure 7:**
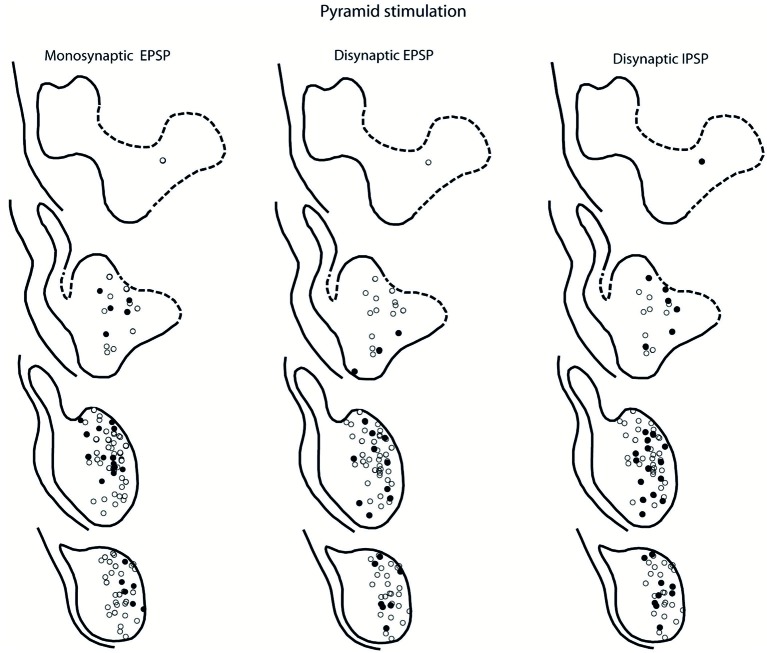
**Location of recorded LRN neurones with contralateral pyramidal input**. Left panels show the location of cells with monosynaptic EPSPs, middle panels show cells with disynaptic EPSPs after C5 DLF transection, and right panels show cells with disynaptic IPSPs after C5 DLF transection. Filled circle are cells with effect and open cells with no effect.

**Figure 8:**
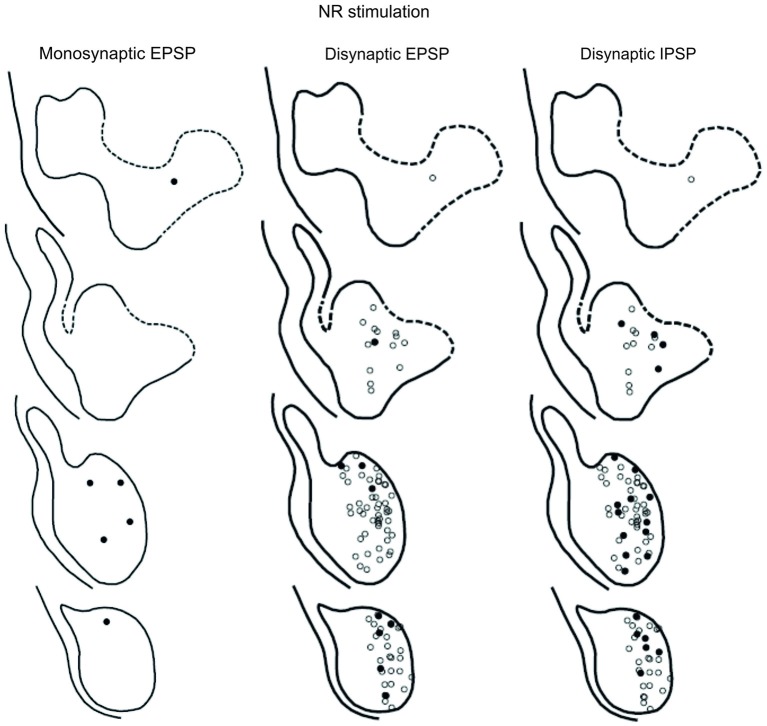
**Location of recorded LRN neurones with contralateral rubral input**. Left panels show the location of cells with monosynaptic EPSPs, middle panels show cells with disynaptic EPSPs after C5 DLF transection, and right panels show cells with disynaptic IPSPs after C5 DLF transection. Filled circle are cells with effect and open cells with no effect.

The location of LRN neurones receiving monosynaptic pyramidal and rubral excitation and disynaptic excitation and inhibition mediated by C3-C4 PNs from the pyramid and NR, is shown in Figures 7 and 8, respectively. The LRN was divided into 1 mm thick segments, the lower being the most caudal section. Recordings were made mainly from the caudal and middle part of the nucleus, which receives most of the input from the spinal cord. In Figures 7 and 8 are shown: left column, the location of cells with monosynaptic excitation; middle column, the location of cells with disynaptic excitation and right column, the location of cells with disynaptic inhibition (filled circles). Empty circles indicate recorded cells lacking synaptic input from these systems.

The LRN cells with monosynaptic pyramidal and rubral excitation were located in the dorso-medial part of the nucleus. In contrast, LRN cells with disynaptic EPSPs, mediated via the C3-C4 PNs, were located both in the dorso-medial part, central and ventro-medial parts of the nucleus. Together these data indicate that the direct pyramidal and rubral modulation of LRN neurones is restricted to the dorso-medial part of the LRN, which was shown to project mainly to the ipsilateral pars intermedia and paramedian lobule V in the cerebellum with input from the iFT (Clendenin et al., 1974c; Ekerot, 1990a). Furthermore, the disynaptic effects mediated via the C3-C4 PNs reached not only this region of the LRN, but also more ventral parts of the nucleus, which was shown to project bilaterally in lobules IV and V of the anterior lobe and in the vermis of lobule VIII with input from the bVFRT (Clendenin et al., 1974b). These findings corroborate earlier observations on the termination of C3-C4 PNs in the LRN (Alstermark et al., 1981a).

### Convergence and Synaptic Integration in Subtypes of LRN Neurones

The large number of inputs to the LRN (monosynaptic input from the pyramid and NR, C3-C4 PNs, iFT, and cervical and lumbar bVFRT) suggest an elaborate process of descending and ascending synaptic integration in LRN neurons before producing mossy fiber output to the cerebellum. This integration has an additional layer of complexity since all spinal afferent systems consist of both excitatory and inhibitory neurones, while the monosynaptic input from the pyramid and NR is only excitatory. To investigate the process of integration, we identified LRN neuron subtypes receiving monosynaptic EPSPs from the contralateral pyramidal (Table 1). Only 5 out of 61 tested LRN neurons received monosynaptic rubral EPSPs and therefore it was not meaningful to make a table of these cells. Disynaptic pyramidal and rubral EPSPs and IPSPs mediated via presumed C3-C4 PNs were commonly observed in the different subtypes of LRN neurons (Table 2).

### LRN Neurons with Monosynaptic Input from Pyramid

Comparison of convergence in single LRN neurones revealed two major differences. First, among LRN neuron subtypes, it was common to receive monosynaptic pyramidal excitation among iFT, bVFRT (Table 1) and C3-C4 PNs (Table 2) in a range of 35–60%. In contrast, monosynaptic pyramidal excitation was rarely observed in LRN neurons with monosynaptic rubral input or from lumbar bVFRT LRN neurons (Table 1). Interestingly, in each LRN neuron with monosynaptic pyramidal excitation, there was no overlap of excitation and inhibition for each of the converging systems shown in Table 1. Thus, motor cortex can select differentially among LRN neurons with excitatory or inhibitory inputs for a given spino-LRN system.

### LRN Neurons with Disynaptic Pyramidal and NR Inputs Mediated via C3-C4 PNs

In all of the investigated LRN neurons there was a broad convergence between the various spino-LRN systems as shown in Table 2. Of particular interest in this study is the fact that LRN neurons with input from presumed C3-C4 PNs evoked from the pyramid and NR, also exhibited converging excitation and/or inhibition from the pyramid, iFT and bVFRT systems. This holds true also for LRN neurons with input from cervical or lumbar bVFRT systems.

## Discussion

It is clear that the spino-LRN-cerebellar route provides information from many spinal pre-motoneuronal centers (cf. review by Alstermark and Ekerot, 2013) and as pointed out in a companion *Perspective article* (Jiang et al., 2015) in this research topic, the iFT, bVFRT and C3-C4 PN systems may reflect a phylogenetic development in need of increased control of dexterous forelimb movements. We first discuss the input from the C3-C4 PN system alone, then the convergence of effects from all three systems and finally functional implications of the convergence patterns.

### C3-C4 PN Modulation of the LRN

**Figure 9:**
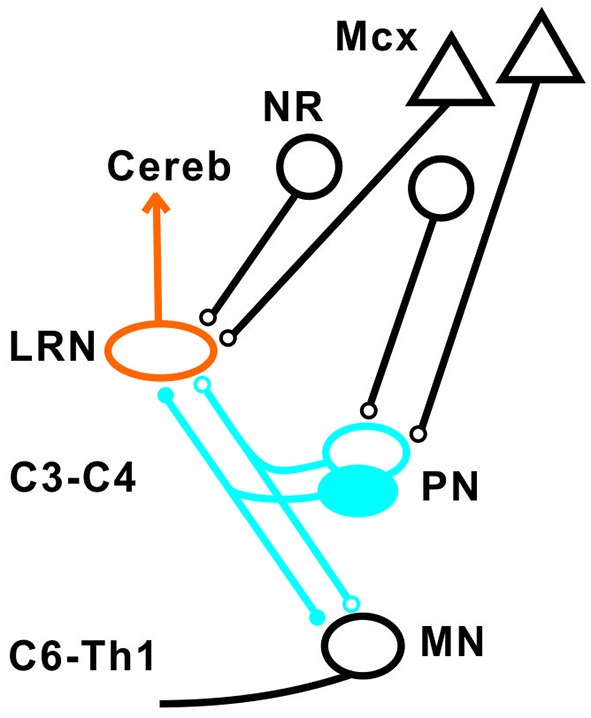
**Summary of direct pyramidal, rubral and indirect pathways via C3-C4 PN to LRN neurons**. Open synapses are excitatory and filled synapses are inhibitory. The arrowhead from the LRN neurons indicated multiple terminations (in deep cerebellar nuclei and granule cell layers) in the cerebellum.

Our results show that disynaptic excitation and inhibition in LRN neurons from cortico- and rubrospinal fibers could be mediated by convergent input to neurons in the C3-C4 segments, as demonstrated by the persistence of disynaptic effects after C5 lesion, but their elimination after C2 lesion. In addition, stimulation of the isolated DLF in C3 (following DLF transection in both C2 and C5) evoked disynaptic EPSPs and IPSPs in LRN neurones at low threshold, also showed transmission via neurons in the C3-C4 segments. It was previously shown that the vast majority (>84%) of the PNs have bifurcating axons projecting to the LRN and MNs (Alstermark et al., 1981a), which is in contrast to medial segmental interneurons (no projection to MNs) recorded in the same segments as the PNs (Alstermark et al., 1984c). These authors found that about 20% of these medial segmental interneurons have ascending collaterals to the LRN. Second, these interneurons, have weak or no convergent excitatory input from NR and tectum (Alstermark et al., 1984c). Since we demonstrate a strong facilitation of pyramidal EPSPs and IPSPs from NR after C5 DLF (Figures 3A,D), these effects must have been mediated via excitatory and inhibitory C3-C4 PNs. Furthermore, a majority of these LRN cells receiving either disynaptic excitation or inhibition via the C3-C4 PNs, also had monosynaptic excitatory inputs from the cortico-reticular neurons, as illustrated in Figure 9.

The projection from motor cortex to the LRN is not via collaterals of corticospinal fibers, but from a separate population of cortico-reticular neurons terminating in the brainstem (Alstermark and Lundberg, 1982; Matsuyama and Drew, 1997). Whether or not the rubro-reticular fibers are collaterals from rubrospinal neurones is not known, but we have tentatively illustrated them as a separate population in Figure 9. Such separate control of LRN neurons from the motor cortex and possibly from the brain stem is interesting functionally, because it indicates a need for higher centers to select among the different subpopulations of spino-LRN-cerebellar neurons. However, the monosynaptic rubro-LRN excitation was observed in only 5% in contrast to almost 60% for the pyramidal-LRN excitation in LRN neurons with disynaptic input mediated via the C3-C4 PNs, suggesting a much smaller impact for a rubro-LRN control on this subpopulation of LRN neurons.

### Convergence of Excitation and Inhibition in LRN Neurones

**Figure 10:**
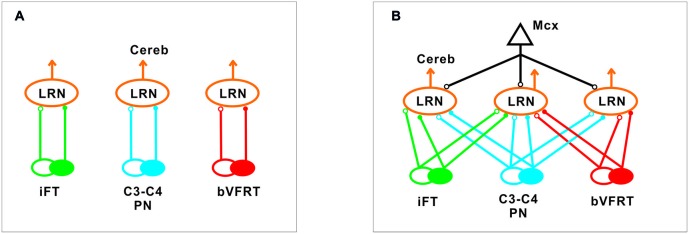
**Comparison of excitation and inhibition evoked from iFT, C3-C4 PN and bVFRT systems in LRN neurons. (A)**, Separate subpopulations of LRN neurons signal information from excitatory and inhibitory iFT, C3-C4 PN and bVFRT systems to the cerebellum. **(B)**, Subpopulations of LRN neurons signal convergent information from excitatory and inhibitory iFT, C3-C4 PN and bVFRT systems in different combinations to the cerebellum. Motor cortex (Mcx) has selective access to the various subpopulations of LRN neurons.

Our results corroborate earlier findings by Ekerot and colleagues that there is a parallel input from excitatory and inhibitory iFT and bVFRT neurons to subpopulations of LRN neurons, and in addition show a similar organization for the C3-C4 PN system as illustrated in Figure 10A.

The first extensive investigation of the synaptic organization of a spino-cerebellar system was performed by Lundberg and Weight (1971) and by Lundberg (1971) on the ventral spino-cerebellar tract (VSCT; see review Baldissera et al., 1981). Their results revealed a complex integration of excitation and inhibition from low threshold muscle afferents, high threshold flexion reflex afferents and descending systems on to VSCT neurones. Based on these results Lundberg (1971) proposed the hypothesis “that some VSCT neurones monitor transmission in inhibitory pathways to motoneurones by measuring the output from last order inhibitory interneurons against the excitatory input to them”.

In higher mammals, like the cat and monkey, it is not known if the iFT and cervical bVFRT systems have direct projections also to motoneurons as the C3-C4 PNs, but recent findings in the mouse show that this is the case (Pivetta et al., 2014). Interestingly, whereas the VSCT neurons mainly receive input from inhibitory interneurons (Jankowska et al., 2010), the LRN neurons receive input from both excitatory and inhibitory interneurons. Another difference is that a majority of LRN neurons receive convergence, excitatory and inhibitory, from at least two of the ascending systems investigated in this study as shown in Figure 10B. A smaller fraction receives convergence from all three systems (iFT, bVFRT and C3-C4 PN). Apparently, there is a possibility for the cerebellum to compare the excitability level of each system alone as well as in combination. Thus, although these indirect spino-cerebellar pathways share several common properties with the direct spino-cerebellar tracts, the difference may be related to the increased demands required to control dexterous forelimb movements (Jiang et al., 2015).

### Function of Indirect Cervical Spino-LRN-Cerebellar Pathways

The goal of reaching is to approach and then grasp an object, and the timing between these two motor components is critical for a successful movement. The role of the C3-C4 PNs is to mediate the command for reaching as has been demonstrated in the cat, monkey and mouse (Alstermark and Lundberg, 1992; Alstermark and Isa, 2012; Azim et al., 2014). Importantly, in the mouse it was shown that the ascending branch from PNs to the LRN involves a cerebellar loop that can affect motoneurons and reaching behavior (Azim et al., 2014). Behavioral studies in the cat have shown that grasping is primarily controlled by interneurons within the forelimb segments (C6-Th1; Alstermark et al., 1981b; Alstermark and Kümmel, 1990). It therefore seems likely that the cerebellum, would need to receive grasping information from these forelimb segmental interneurons in conjunction with information regarding reaching mediated by C3-C4 PNs. Last-order segmental interneurons within the forelimb segments have been identified that mediate disynaptic corticospinal excitation to forelimb motoneurones (Alstermark and Sasaki, 1985; Sasaki et al., 1996). One possibility is that at least a subset of the segmental interneurons involved in the control of grasping are included in the iFT system which sends information from forelimb segments to the LRN. As shown in Table 2, excitatory and inhibitory iFT convergence was commonly found in LRN neurones with input from presumed C3-C4 PNs.

**Figure 11:**
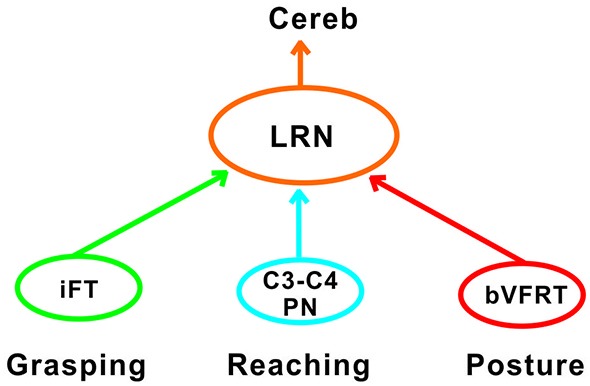
**Comparison of functions at systems level in the LRN**. Information about grasping, reaching and posture can be mediated via the iFT, C3-C4 PN and bVFRT systems to the LRN. In the LRN, a comparison can be made about the activity level of each system in isolation, but also their combined effects. The overview can then be further analyzed in the deep cerebellar nuclei and cerebellar cortex.

Another requirement for successful reaching and grasping is concomitant postural control, especially in the contralateral forelimb that supports much of the body weight (Alstermark and Wessberg, 1985). Previous experiments using activity-dependent transneuronal uptake of WGA-HRP into last-order interneurones to identify spinal circuits involved in reaching and grasping revealed not only C3-C4 PNs and segmental interneurones on the ipsilateral side of injection, but also commissural interneurones in the forelimb segments on the contralateral side (Alstermark and Kümmel, 1990). Given their location, we propose that some of these contralateral neurones belong to the cervical and lumbar bVFRT systems, providing ascending information about the coordination of the limbs. Convergence from these systems was often found in LRN neurons with input from presumed C3-C4 PNs (Table 2). Taken together, as shown schematically in Figure 11, we propose that the LRN may provide an overview of reaching, grasping and posture to cerebellum that could compare the activity to make fast updating and corrections by the use of the internal feedback from the various spino-LRN-cerebellar pathways.

## Author Contributions

BA and C-FE contributed equally to the work.

## Conflict of Interest Statement

The Guest Associate Editor Dr. Lan declares that, despite having collaborated with the author Dr. Alstermark, the review process was handled objectively and no conflict of interest exists. The authors declare that the research was conducted in the absence of any commercial or financial relationships that could be construed as a potential conflict of interest.
